# Child Wasting in Emergency Pockets: A Meta-Analysis of Small-Scale Surveys from Ethiopia

**DOI:** 10.3390/ijerph13020178

**Published:** 2016-01-28

**Authors:** Chiara Altare, Tefera Darge Delbiso, Debarati Guha-Sapir

**Affiliations:** Centre for Research on the Epidemiology of Disasters, Institute of Health and Society, Université catholique de Louvain, Clos Chapelle-aux-Champs, 30.94, 1200 Brussels, Belgium; tefera.delbiso@uclouvain.be (T.D.D.); debarati.guha@uclouvain.be (D.G.-S.)

**Keywords:** wasting, small-scale surveys, meta-analysis, Ethiopia

## Abstract

Child undernutrition is a major public health concern in Ethiopia (stunting national prevalence: 44%; wasting: 10%), despite the overall improvement in child health status during the last decade. Hundreds of small-scale surveys are conducted in Ethiopia’s emergency pockets under ENCU’s supervision. We reviewed the evidence from small-scale surveys conducted between 2008 and 2013 with two objectives: to provide a summary estimate of wasting prevalence from emergency pockets and to examine reasons for variation in prevalence estimates. We created a dataset by combining data from the Complex Emergency Database, the Famine Early Warning System Network and the Armed Conflict Location Event Data. We conducted a meta-analysis of small-scale surveys using a random effects model with known within-study heterogeneity. The influence of survey covariates on estimated prevalence was investigated with meta-regression techniques. We included 158 surveys in the analysis. A high degree of heterogeneity among surveys was observed. The overall estimate of wasting prevalence was 10.6% (95% CI 9.8–11.4), with differences among regions and between residents and refugees. Meta-regression results showed that vaccination coverage, child mortality, diarrhea prevalence and food insecurity are significantly associated with wasting prevalence. Child care and displacement status were not. Aggregated analysis of small-scale surveys provides insights into the prevalence of wasting and factors explaining its variation. It can also guide survey planning towards areas with limited data availability.

## 1. Introduction

Child undernutrition is a major public health concern in Ethiopia, despite the overall improvement in child health during the last decade. Between 2000 and 2011, infant mortality declined from 97 deaths per 1000 live births to 59 and child mortality from 77 down to 31. In terms of nutritional achievements, stunting among children under five dropped from 58% down to 44%, underweight from 41% to 29% and wasting from 12% to 10%. However, Ethiopia still ranks worst among Eastern African countries as far as both stunting and wasting are concerned [1]. Ethiopia is facing a chronic crisis with a high prevalence of stunting and developmental challenges. It also has emergency pockets, where wasting threatens child survival. According to WHO’s classification of the severity of malnutrition [2], a prevalence of acute malnutrition of 10% is considered as serious. However, the nationwide estimate masks important regional differences: wasting prevalence ranges from 4% in Addis Ababa to 22% in the Somali region [1].

Extensive research has shown the short- and long-term brunt of undernutrition: increased mortality and morbidity risk, poorer mental and psychomotor development and loss of human capital [3,4,5]. Numerous studies have investigated the causes of undernutrition, which have been regrouped into three levels (basic, underlying and immediate), and encompass three areas: food security and adequacy; child care; access to health services and health status [6]. Studies from Ethiopia have shown that undernutrition is associated with household food insecurity and poor eating habits [7,8], inadequate feeding practices, poor access to health services and poor sanitation [9,10,11]. 

Besides data collected for specific research studies, a variety of nutrition data exist from Ethiopia that are managed by the Disaster Risk Management and Food Security Sector (DRMFSS) within the Ministry of Agriculture. The Emergency Nutrition Coordination Unit (ENCU) was created within the DRMFSS in 2000 to strengthen the early warning system in nutrition surveillance and response. ENCU coordinates emergency nutrition surveys that are conducted with national and international non-governmental organizations based on national guidelines [12]. The emergency nutrition surveys provide representative results from the district (*woreda*) where they are conducted. Although the contribution of each survey by itself is limited by its small sample size and the impossibility to extrapolate results to the national level, their aggregation can summarize recent evidence from emergency areas. 

To our knowledge, there has been no attempt to consolidate the results from these small-scale surveys to derive a robust prevalence estimate of wasting from emergency pockets in Ethiopia and to assess how this is influenced by various factors. Investigating how prevalence varies according to features, such as child care or health status, offers insights into possible etiology. The aim of the paper is thus two-fold: first, to provide a summary estimate of wasting prevalence from emergency pockets and, second, to examine reasons for variation in prevalence estimates by investigating characteristics at the survey level with meta-regression techniques.

## 2. Materials and Methods 

### 2.1. Dataset

We combined data from mainly three sources and created the study dataset. These are: the Complex Emergency Database (CEDAT) [13], the Famine Early Warning System Network (FEWSNET) [14] and the Armed Conflict Location Event Data (ACLED) [15]. The study dataset includes 158 observations over the period of 2008–2013, from nine out of eleven Ethiopian regions. The unit of analysis is the survey, as survey reports and not survey datasets are compiled in CEDAT. 

### 2.2. Search Strategy and Inclusion Criteria

The Complex Emergency Database was searched for surveys conducted in Ethiopia between 2008 and 2013 and reporting the following items: prevalence of global acute malnutrition (GAM), GAM confidence interval, GAM sample size, sampling design, population status, location and time of data collection. The ENCU list of conducted surveys was also screened, and reports not already available in CEDAT were requested from the implementing agency. The survey methodology was reviewed, and surveys that used purposive sampling were discarded as not representative of the surveyed population. Included surveys used multistage cluster sampling or exhaustive sampling. Wasting prevalence is measured with GAM among children between 6 and 59 months, and defined as a −2 or less weight-for-height (WFH) z-score according to the WHO standard population and/or edema. Historical survey estimates based on the NCHS growth reference have been converted to WHO-based prevalence using an established algorithm [16].

### 2.3. Data Extractions and Variable Definition

The following data were extracted from survey reports: prevalence of GAM, prevalence of chronic malnutrition, CMR, U5MR (both expressed in deaths/10,000/day), measles-containing vaccine (MCV) coverage, occurrence of diarrhea events in the two weeks before the survey, early initiation of breastfeeding (EIB; as defined by WHO [10]), location (at the third administrative level), time (month and year in which the survey was conducted), displacement status (residents or refugees), sample size, confidence intervals and survey sampling method. Information on the severity of food insecurity based on the Integrated Food Security Phase Classification (IPC) at the time and area of each survey was extracted from monthly update reports from the Famine Early Warning System Network. We created an “insecurity-exposure rate” by combining conflict events from the ACLED database and population estimates from the most recent census [17], using a population growth rate of 2.1% [18]. We also created a variable “NGO presence” from the “Who What Where” (3W) database from the UN Office for the Coordination of Humanitarian Affairs (OCHA) to control for humanitarian interventions. Table 1 defines the variables used in the analysis and how they were measured. 

### 2.4. Assessment of Data Quality and Completeness

Point estimates of GAM, CMR, U5MR and MCV were verified by recalculating them with data provided in the reports. If data were not available, the accuracy of reporting was confirmed comparing the value in the report and in CEDAT. Four variables reported missing values: MCV (complete 97.5%), diarrhea (complete 92.4%), early initiation of breastfeeding (complete 59.5%) and chronic malnutrition (complete 60.1%). Overall, dataset entries were complete at 92.5%. We used hot deck imputation, which involves replacing missing values with values from a similar responding unit [19], in our case a survey from the same third administrative unit (when more than one survey existed from the same *woreda*, the mean value was taken; when no surveys existed from a given *woreda*, the mean of the second administration level (Admin2) was used, and so on, going up in the administrative division scale). Missing EIB values originated mainly from older surveys and were distributed among all regions. MCV values that we coded as missing were available in the survey report, but used inappropriate denominators (6–59 months instead of 9–59 months). Diarrhea occurrence was not available in 12 survey reports from refugee camps. Missing stunting values did not show any distinct pattern. 

### 2.5. Statistical Analysis

We conducted a meta-analysis of small-scale nutrition surveys. We used a random effects model with known within-study heterogeneity, which allows for the generalization of results from numerous surveys [20]. Variances of point estimates were calculated from the 95% confidence intervals as in [21]. Design effects were estimated from the confidence intervals and included in the variance calculation. A finite population correction was applied to the variance. 

The response variable (GAM) was log-transformed because the prevalence of global acute malnutrition is positively skewed. The variance of a log-transformed variable was calculated using the delta method [22]. Variation and estimates of wasting prevalence were calculated for the entire dataset, by region and by displacement status.

**Table 1:** Definition of variables used in the analysis. OCHA, Office for the Coordination of Humanitarian Affairs; 3W, “Who What Where”.

Variable Acronym	Variable Name	Variable Definition and Measurement	Source
GAM	Prevalence of global acute malnutrition in children aged 6–59 months.	GAM is defined as WFH < −2 standard deviations from the median weight of the standard distribution for children of the same height and/or having edema. It is expressed as the prevalence (%) and calculated as n/N × 100, where n is the number of wasted children and N the sample size.	Survey report
Chronic Malnutr.	Prevalence of chronic malnutrition in children aged 6–59 months	Chronic malnutrition is defined as height for age < −2 standard deviations from the median height of the standard distribution of children of the same age. It is expressed as the prevalence (%).	Survey report
U5MR	Under five mortality rate	The under five mortality rate is calculated as the number of deaths among children under the age of 5 over a given period of time divided by an estimate of the population at risk of dying during that period. It is expressed as 10,000/day.	Survey report
MCV	Measles-containing vaccine	Proportion of children (9–59 months) vaccinated against measles over the children in the appropriate age group eligible for vaccination. Vaccination status is based both on the vaccination card and mother’s recall. It is expressed as prevalence.	Survey report
EIB	Early initiation of breastfeeding	Proportion of children aged 0–24 months who were breastfed within 1–2 h after birth [10].	Survey report
Diarrhea	Occurrence of diarrhea in children aged 6–59 months	Proportion of children reporting diarrhea (or watery diarrhea in case a distinction between watery and bloody was made) in the previous 2 weeks. Diarrhea is defined as at least 3 events per day.	Survey report
FS	Level of food security	Using the IPC classification, regions are classified as follows: 1 = none/minimal food insecurity 2 = stressed 3 = crisis 4 = emergency 5 = famine	FEWSNET Update
C_admin3_2m	Exposure to insecurity events	Rate of exposure to insecurity events at the Admin3 level. This is calculated as the number of events occurring in the month in which the survey was conducted and in the previous one, over the population at risk per 100,000.	Created using ACLED and census
Hump	Humanitarian presence	Number of national and international NGOs providing services in Admin3	OCHA 3W + survey reports
Admin1	First administrative level	Region	Survey report
Admin2	Second administrative level	Zone	Survey report
Admin3	Third administrative level	*Woreda*	Survey report
City		City	Survey report
Camp		Refugee camp	Survey report
Discat	Displacement category	1 = resident 2 = refugee	Survey report
Geographical area	Area within Ethiopia	1 = Afar, Amhara, Benishangul-Gumuz, Tigray (north) 2 = Oromia, Gambella, SNNP (south) 3 = Somali, Dire Dawa (east)	Created

The potential influence of covariates on the prevalence estimates was investigated using meta-regression, first through bivariate analyses and then through a multivariate analysis to investigate which variables were associated with wasting if there were adjustment of other study covariates. Multivariate models were based on UNICEF’s malnutrition framework [6] and included:

(1) Underlying factors:

- MCV coverage, as a proxy for the availability of health services;
- Child mortality rate, as a proxy for the health status of the population;
- Early initiation of breastfeeding, as an indicator of the adequacy of child care;
- Level of food (in)security;
- Prevalence of diarrhea, as a proxy for water and sanitation conditions.

(2) Basic factors:

- Region of residence;
- Displacement status;
- Exposure to insecurity;
- Presence of humanitarian interventions;
- Stunting level, as a proxy for deprivation chronicity.

The goodness-of-fit of the models was assessed using the log-likelihood test. Multicollinearity between predictor variables was checked using the variance inflation factor (VIF) (a cutoff of 5 was applied). All tests were two-sided, and a *p* < 0.05 was considered statistically significant. The analysis was conducted in R (Version 3.2.1) [23] using the Metafor package (Version 1.9-7) [24].

## 3. Results

### 3.1. Characteristics of the Surveys

A total of 160 surveys was retrieved: two were duplicates and were excluded. We therefore included 158 surveys in the analysis. It was possible to validate all but five point estimates of GAM, and no error was found. The majority of the surveys (114) reported GAM prevalence based on WHO standards, while 44 surveys only displayed prevalence based on the NCHS reference. 

**Figure 1:**
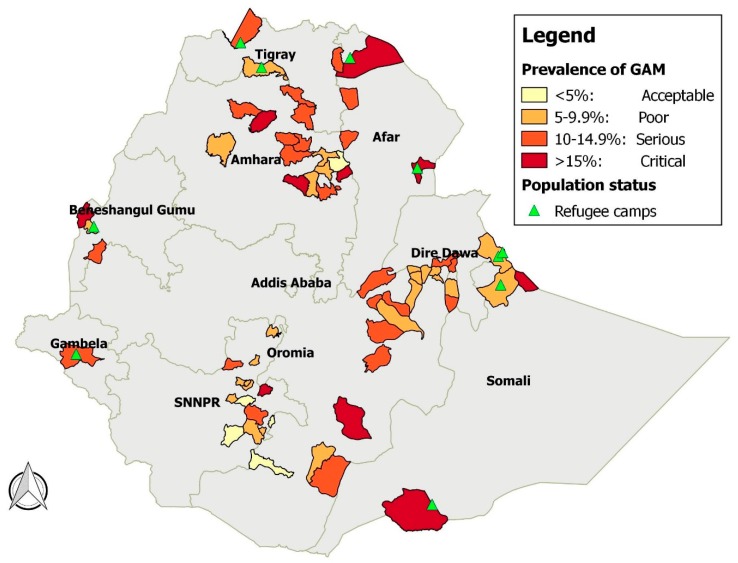
Prevalence of GAM by district, Ethiopia, 2008–2013.

The surveys come from nine regions (Figure 1) and are representative of around 8.5 million people. They include both residents (74% of the surveys) and refugees (26%). Table 2 shows the number of surveys by state and population group. No survey originates from Addis Ababa and Harari, because these are predominantly urban settings, while emergency nutrition surveys are mainly conducted in rural areas with serious malnutrition. Refugee populations include Somalis in the Somali region, South Sudanese in Gambella and Benishangul-Gumuz and Eritrean in Tigray and Afar. The majority of the surveys originate from the most populated regions (Oromia, Amhara and Southern Nations and Nationalities People (SNNP)). Somali, Benishangul-Gumuz and Afar regions are overrepresented due to the presence of refugees. The survey samples ranged from 140–976 children aged 6–59 months. The mean sample size is 623.

**Table 2:** Survey distribution by region and population status, Ethiopia emergency pockets, 2008–2013.

Regions	Resident	Refugee	Total # of Surveys	% of the Total Surveys	Population Census 2007	% of the Total Population
Afar	3	4	7	4.4	1,415,370	2
Amhara	26	0	26	16.5	18,968,100	26.8
Benishangul-Gumuz	2	6	8	5.1	625,090	0.9
Dire Dawa	2	0	2	1.3	370,100	0.5
Gambella	0	5	5	3.2	245,592	0.4
Oromia	49	0	49	31.0	26,623,230	37.6
SNNP	32	0	32	20.3	14,566,820	20.6
Somali	2	20	22	13.9	3,714,740	5.2
Tigray	1	6	7	4.4	4,299,700	6
Total	117	41	158	100	70,828,742	100

### 3.2. Description of Heterogeneity among Studies and Summary of Prevalence

There was wide variation in the prevalence estimates of wasting (Q = 4018.85, df = 157, *p* < 0.001, I^2^ = 93.74%). The back-transformed mean of the random effects distribution of studies was 10.6% (95% CI: 9.8–11.4).

**Figure 2:**
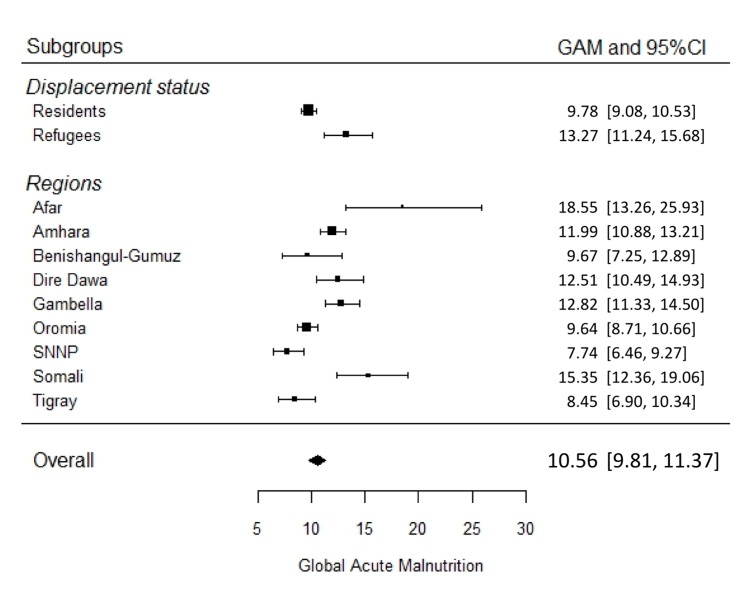
Forest plot of GAM summary estimates of surveys from Ethiopia emergency areas by region and population group, 2008–2013 (prevalence and 95% confidence interval).

Summary estimates by region are shown in the forest plot (Figure 2). Afar and Somali regions reported the highest levels of GAM (18.6% and 15.3%), both above the 15% emergency threshold. SNNP, Tigray, Oromia and Benishangul-Gumuz report values below 10%. Heterogeneity among GAM prevalence is highest in Afar and Somali regions (H^2^ = 27.4 and H^2^ = 35.2, respectively). In Afar, this variability is linked to differences between prevalence among refugees and among residents (twice as high among refugees). In the Somali region, on the other hand, three surveys reported very high GAM values (32.7%, 47.8% and 50.6%). These originated from refugees fleeing the famine in Somalia in 2011–2012. 

Analysis disaggregated by population group shows that wasting prevalence among refugees is statistically higher than among residents: 13.3% (95% CI: 11.2–15.7) *vs.* 9.8% (95% CI: 9.1–10.5) (Figure 2). Furthermore, variability among refugees (Q = 1874.66, df = 40, *p* < 0.0001, I^2^ = 96.78%) is also higher than among residents (Q = 927.79, df = 116, *p* < 0.0001, I^2^ = 89.85%).

### 3.3. Investigation of Sources of Heterogeneity

The associations between study covariates and prevalence estimates from bivariate meta-regression analyses are shown in Table 3, for example wasting prevalence is on average 38% higher among refugees. Displacement status explains 10.4% of the among-studies variability. Among the time factors, year and season do not explain any variability among prevalence estimates. 

Three cases of multicollinearity were identified among variables within the same group: among the health-related variables, it was found between “CMR” and “U5MR” (VIF respectively of 7.7 and 7.1); among the insecurity variables, it was found between “c_admin3_1m” and “c_admin3_2m” (VIF respectively of 8.7 and 8.3); finally, among variables indicating humanitarian presence, multicollinearity was identified between “humanitarian NGOs” and “humanitarian sectors” (VIF respectively of 5.2 and 4.8). This did not represent a problem, as only one variable from each group was foreseen in the final models. VIF in multivariate analysis models was below four.

Table 4 presents the results from the multivariate random effects meta-regression models. Model 1 includes all covariates of interests (underlying and basic factors); Model 2 includes only the significant covariates from Model 1; Model 3 includes the first administrative division instead of the aggregated variable “geographical area”. Model 2 is the best fit of the data. 

**Table 3:** Results of meta-regression of nutrition surveys, Ethiopia emergency pockets, 2008–2013, bivariate analysis.

Variable Group	Variable/Category	Exp (Coefficient) * (95% CI)	*p*-Value	Variance Explained (%)
Displacement	Refugee	1.38 (1.17–1.62)	<0.0001	10.4
Region	Afar	1.94 (1.41–2.66)	<0.0001	26.9
	Amhar	1.24 (1.02–1.51)	0.03	
	Benishangul-Gumuz	1.01 (0.74–1.38)	0.96	
	Dire Dawa	1.31 (0.74–2.33)	0.36	
	Gambella	1.34 (0.92–1.94)	0.13	
	Oromia	Reference		
	SNNP	0.82 (0.68–0.99)	0.04	
	Somali	1.61 (1.32–1.98)	<0.0001	
	Tigray	0.84 (0.60–1.18)	0.31	
Area	North	1.29 (1.11–1.51	0.001	17.4
	South	Reference		
	East	1.68 (1.38–2.04)	0.0001	
Livelihood	Cropping	Reference		16.7
	Agro-pastoral	1.07 (0.87–1.30)	0.5218	
	Pastoral	1.66 (1.37–1.99)	<.0001	
Year	2008	Reference		<0.1
	2009	0.90 (0.70–1.16)	0.41	
	2010	0.85 (0.66–1.1)	0.21	
	2011	1.1 (0.81–1.50)	0.55	
	2012	0.94 (0.70–1.28)	0.70	
	2013	0.98 (0.75–1.27)	0.85	
Month	January	Reference		8.3
	February	1.03 (0.71–1.49	0.89	
	March	1.44 (1.01–2.06)	0.04	
	April	1.38 (0.96–1.99)	0.09	
	May	1.61 (1.05–2.49)	0.03	
	June	1.41 (0.96–2.07)	0.08	
	July	1.32 (0.88–1.99)	0.18	
	August	1.23 (0.83–1.84)	0.31	
	September	1.11 (0.75–1.64)	0.60	
	October	1.87 (1.26–2.78)	0.01	
	November	1.22 (0.80–1.86)	0.36	
	December	1.03 (0.67–1.59)	0.88	
Hunger season	During	1.05 (0.91–1.22)	0.49	<0.1
Health	MCV	0.99 (0.99–1.00)	0.35	<0.1
	Vitamin A	0.99 (0.99–1.00)	0.261	0.1
	CMR	2.34 (1.79–3.06	<0.0001	22.9
	U5MR	1.38 (1.27–1.51)	<0.0001	28.2
	Cough	1.03 (1.00–1.05)	0.01	5.0
Water and sanitation	Diarrhea	1.03 (1.01–1.04)	<0.0001	12.7
	Water	0.99 (0.99–1.00)	0.28	<0.1
Care	EIB	0.99 (0.98–0.99)	0.0007	7.1
Food Security	Food secure	Reference		10.1
	Stressed	1.17 (0.90–1.54	0.25	
	Crisis	1.17 (0.90–1.54	0.24	
	Emergency	2.56 (1.62–4.05)	<0.0001	
Insecurity	c_admin2_1m	2.22 (0.96–5.12	0.06	1.6
	c_admin2_2m	2.41 (1.32–4.39)	0.05	5.0
	c_admin3_1m	1.40 (1.00–1.93)	0.04	2.1
	c_admin3_2m	2.05 (1.11–3.77)	0.02	2.9
Stunting		0.99 (0.98–1.00)	0.14	0.7
Humanitarian presence	Humanitarian NGOs	1.05 (1.03–1.08)	<0.0001	12.9
	Sectors	1.06 (1.03–1.09)	0.0001	9.9

* *e* to the power of the regression coefficient.

**Table 4:** Results of multivariate meta-regression analysis, Ethiopia emergency pockets, 2008–2013.

Covariate	Model 1			Model 2			Model 3		
tau^2^	0.0983			0.0972			0.0935		
tau	0.3135			0.3118			0.3058		
I^2^	86.80%			86.90%			86.40%		
H^2^	7.6			7.6			7.4		
R^2^	52.10%			52.62%			54.40%		
	Exp(coeff.) ^†^	95% CI	*p*-value	Exp(coeff.) ^†^	95% CI	*p*-value	Exp(coeff.) ^†^	95% CI	*p*-value
Intercept	6.88	4.2–11.2	<0.0001	7.94	5.89–10.9	<0.0001	9.52	6.83–13.3	<0.0001
Refugees	0.90	0.70–1.16	0.422						
MCV *	0.9935	0.989–0.997	0.003	0.9935	0.991–0.997	<0.0001	0.9944	0.991–0.998	0.002
EIB	1.00	0.99–1.01	0.365						
FS: Stressed	1.45	1.15–1.82	0.001	1.45	1.17–1.80	0.001	1.17	0.90–1.52	0.250
FS: Crisis	1.33	1.05–1.69	0.018	1.33	1.06–1.67	0.012	1.05	0.80–1.38	0.709
FS: Emergency	2.05	1.31–3.20	0.002	2.19	1.43–3.35	0.000	1.64	1.04–2.60	0.035
Diarrhea	1.02	1.01–1.04	0.002	1.02	1.01–1.03	<0.0001	1.03	1.01–1.04	0.000
U5MR	1.20	1.08–1.35	0.001	1.17	1.06–1.29	0.002	1.19	1.08–1.33	0.001
Insecurity	0.97	0.59–1.59	0.911						
Stunting	1.00	0.99–1.01	0.737						
Hum. NGOs	1.02	0.99–1.04	0.182						
North	1.53	1.30–1.81	<0.0001	1.44	1.26–1.65	<0.0001			
East	1.59	1.19–2.12	0.002	1.63	1.36–1.95	<0.0001			
Amhara							1.49	1.24–1.79	<0.0001
Afar							1.29	0.97–1.73	0.082
Benishangul-Gumuz							0.94	0.66–1.34	0.741
Dire Dawa							1.21	0.75–1.94	0.434
Gambella							1.01	0.71–1.44	0.950
SNNP							0.87	0.73–1.02	0.093
Somali							1.56	1.26–1.94	<0.0001
Tigray							1.01	0.72–1.42	0.963

* Coefficient and CI with 4 and 3 decimals necessary to show the marginal effect of MCV. ^†^ *e* to the power of the regression coefficient.

Variables capturing health factors (MCV and U5MR) and sanitation conditions are consistently significant in the three models. From Model 2, all other variables kept constant, a one percentage point increase in MCV coverage is associated with a 0.65% lower GAM. Given that the response variable is log-transformed, coefficients have a multiplicative effects on the response variable in the case of changes in X other than an increase of one unit. A one-unit increase in the child mortality rate is associated with 17% higher GAM. A one percentage point increase in the prevalence of diarrhea is associated with a 2.3% higher GAM. All levels of food insecurity are significant in Models 1 and 2: areas with stressed food security are associated with 45% higher GAM, whereas GAM is more than double in emergency areas. Only the highest level of food insecurity is significant in Model 3 and is associated with 64% higher GAM. In terms of geographical variation, Amhara (north area) and Somali regions (eastern area) are associated with higher wasting prevalence. Displacement status, early initiation of breastfeeding, exposure to insecurity, chronic malnutrition and humanitarian presence are not significant. 

## 4. Discussion

We conducted a meta-analysis of more than 150 small-scale nutrition surveys collected over a six-year period to summarize evidence from Ethiopian emergency pockets, thereby exploiting a valuable source of recent data. As a comparison, over the same period, we identified a dozen published studies investigating factors associated with wasting that could have been included in a similar meta-analysis [7,9,10,25,26,27,28,29,30,31,32,33]. 

When comparing the results of our analysis with the 2011 DHS [34] (Table 5), we note that the prevalence of wasting among resident population in emergency pockets (9.8%) is similar to the nationwide prevalence (9.7%). Prevalence estimates similar to the DHS also originate from Amhara, Dire Dawa, Oromia and SNNP, regions where only residents were surveyed. This may either indicate that the situation is serious not only in the so-called emergency pockets, but in the entire region; or that the emergency factors that triggered the implementation of a survey are poorly captured by the prevalence of wasting. The few surveys available from residents in Afar and Somali regions report lower prevalence than in the DHS. Emergency interventions may be missing a substantial part of the population living in regions with refugees, as the attention may focus on camps. The analysis of the distribution of surveys may facilitate the targeting of areas where data are needed (for example, residents in Afar, Benishangul-Gumuz and Somali) and guide survey planning.

The covariates most strongly associated with prevalence estimates are health and sanitation factors. Our analysis shows that higher vaccination coverage is associated with lower wasting prevalence. Vaccination coverage seems a good operational predictor of malnutrition. This highlights the importance of having some measure of vaccination coverage for planning aid programs. Child immunization is part of the governmental Health Extension Program [35] launched in 2003 to boost coverage of preventive and basic curative interventions. Monitoring annual increases in coverage can provide useful information on the expected wasting prevalence. The association between wasting and child mortality should be seen in light of the relationship between malnutrition and disease burden. As undernutrition rises exponentially the risk of dying by increasing case fatality of common childhood infections [36], it is no surprise that wasting and high child mortality often occur in the same population. An increase in child mortality, even when the causes are not clear, should prompt nutrition interventions.

**Table 5:** Comparison of wasting prevalence between DHS 2011 and emergency pockets, by region and population group.

Region	Wasting Prevalence (%)		
	DHS 2011 (95% CI)	Emergency Pockets (95% CI)	
	Residents	Residents	Refugees
Overall	9.7 (8.7–10.7)	9.8 (9.1–10.5)	
Amhara	9.9 (7.7–12.1)	11.9 (10.9–13.2)	
Dire Dawa	12.3 (9.0–15.5)	12.5 (10.5–14.9)	
Oromia	9.7 (7.9–11.5)	9.6 (8.7–10.7)	
SNNP	7.6 (6.1–9.2)	7.7 (6.5–9.3)	
Afar	19.5 (16.9–22.1)	11.5 (10.0–13.1)	26.2 (22.5–30.6)
Benishangul-Gumuz	9.9 (7.6–12.1)	14.7 (10.8–19.9)	8.4 (6.2–11.2)
Somali	22.2 (18.3–26.0)	17.3 (15.0–20.0)	15.2 (12.0–19.3)
Gambella	12.5 (8.4–16.7)		12.8 (11.3–14.5)
Tigray	10.3 (8.3–12.3)	6 (4.1–8.6)	9.6 (8.6–10.8)

Diarrhea during the previous two weeks was associated with higher levels of wasting. Their association has been shown to be bidirectional: diarrhea can cause undernutrition through a loss of nutrient intake, malabsorption and the effect of the inflammatory process [37]; on the other hand, malnutrition may also increase the severity of diarrhea, as it leads to reduced immunity and, therefore, to frequent enteric infections [4]. Since 60% of Ethiopian rural households still do not have access to improved water sources [34], strengthening strategies to increase the provision of water and sanitation interventions is crucial for the health of pre-school children. 

Food security also explains part of the heterogeneity among wasting prevalence. A deeper look at the data shows that this association holds only among refugees: populations from famine-affected areas in Somalia resettled in neighboring Ethiopia, also suffering extreme food insecurity. On the contrary, we did not find any association between food security and wasting prevalence among residents (results not shown). This seems surprising in a country that experienced drought in four out of the six years under analysis [38]. However, it may reflect an overall poor diet variety across livelihood and regions that is more related to cultural habits than to effective availability or access to food [39]. A better understanding of the food security situation at the household level is necessary and could be achieved by collecting a set of standardized indicators in small-scale surveys (for example, the household diet diversity score or the coping strategy index [40,41]). 

Displacement status did not contribute in explaining heterogeneity in the multivariate analysis, although the summary estimate for refugees was statistically higher than for residents. Three very high prevalence estimates from refugees in the Somali region drive this difference. We can conclude that refugees are worse off than residents only in Afar, while in Somali and Benishangul-Gumuz, the confidence intervals of prevalence estimates overlap (Table 5). The difference in health status between residents and refugees is often a function of the time spent in the camp. Upon arrival, refugees have shown dire health conditions in many crises [42], which improved thanks to the humanitarian assistance provided in the camp. In certain cases, refugees have been better off than local communities [43]. 

We did not identify any association between child care and wasting. While adequate child care is recognized as an essential element for child growth, the association between feeding practices and wasting is spurious, as it varies by indicator used [10]. We used early initiation of breastfeeding as a proxy for adequate care, since it was available in the majority of the survey reports and is estimated for all children 0–24 months in the sample. Other indicators of infant and young child feeding practices are based on much smaller age groups (for example, continued breastfeeding at one year is calculated only for children between 12 and 15 months; time of weaning is calculated only for children between six and eight months), making these estimates too imprecise. However, EIB can be affected by recall bias, while more recent practices may be easier to remember. It would be useful to collect information for calculating composite indexes as suggested by WHO or information on maternal education, which is widely accepted to be associated with improved child health and nutrition outcomes [44]. 

### Study Limitations

First, the dataset compiles secondary data. It was impossible to verify the application of survey methods, as well as the quality of anthropometric measurements, as primary data were not available. The majority of the surveys used, however, the SMART (Standardized Monitoring and Assessment of Relief and Transition) methodology [45], which provides a standardized and validated method for nutrition surveys, now well established among humanitarian organizations. Second, biases cannot be completely excluded. As survey locations were most likely biased towards areas where undernutrition levels were suspected to be particularly problematic, results may not apply to more nutritionally-secure areas and cannot be generalized to the entire country. Furthermore, we cannot exclude the possibility that some surveys were not captured and, thus, not included in the analysis. As all major international NGOs are regular survey providers to CEDAT and were contacted to obtain reports, we believe this number is negligible. Finally, meta-analysis can be affected by ecological fallacy, *i.e.*, relating the results from across studies to participants’ characteristics within studies. Unfortunately, without individual data (like in our case), this cannot be investigated. 

## 5. Conclusions

This review shows that the aggregated analysis of small-scale surveys provides insights into the prevalence of wasting, identifies factors explaining its variation and can guide survey planning towards areas with limited data availability. Replicating such an analysis in other countries at regular intervals would increase the return of investment in data collection, going beyond the usefulness of each individual survey.

Health and sanitation factors explain the majority of variation among wasting prevalence, while food security, child care and displacement status are not or only marginally associated with wasting. Increased standardization of food security and child care data would improve the comparability of survey results, as well as the understanding of the contextual factors associated with wasting in emergency areas.

## Abbreviations

The following abbreviations are used in this manuscript:

ACLED: Armed Conflict Location Event Data
CEDAT: Complex Emergency Database
CMR: crude mortality rate
DHS: Demographic and Health Survey
DRMFSS: disaster risk management and food security sector
EIB: early initiation of breastfeeding
ENCU: Emergency Nutrition Coordination Unit
FEWSNET: Famine Early Warning System Network
FS: food security
GAM: global acute malnutrition
IPC: Integrated Food Security Phase Classification
MCV: measles-containing vaccine
NCHS: National Centre for Health Statistics
SNNP: Southern Nations and Nationalities People
U5MR: under 5 mortality rate
VIF: variance inflation factor
WHO: World Health Organisation
